# Supplementary material for: Deep Sequencing of Small RNAs from Neurosurgical Extracellular Vesicles Substantiates miR-486-3p as a Circulating Biomarker that Distinguishes Glioblastoma from Lower-Grade Astrocytoma Patients
Source: Int J Mol Sci. 2020 Jul 13;21(14):4954. doi: 10.3390/ijms21144954 (PMC7404297; doi:10.3390/ijms21144954)
Supplement: Supplementary file 1 [file ijms-21-04954-s001.zip › Supplementary Material/Supplementary Material.docx]

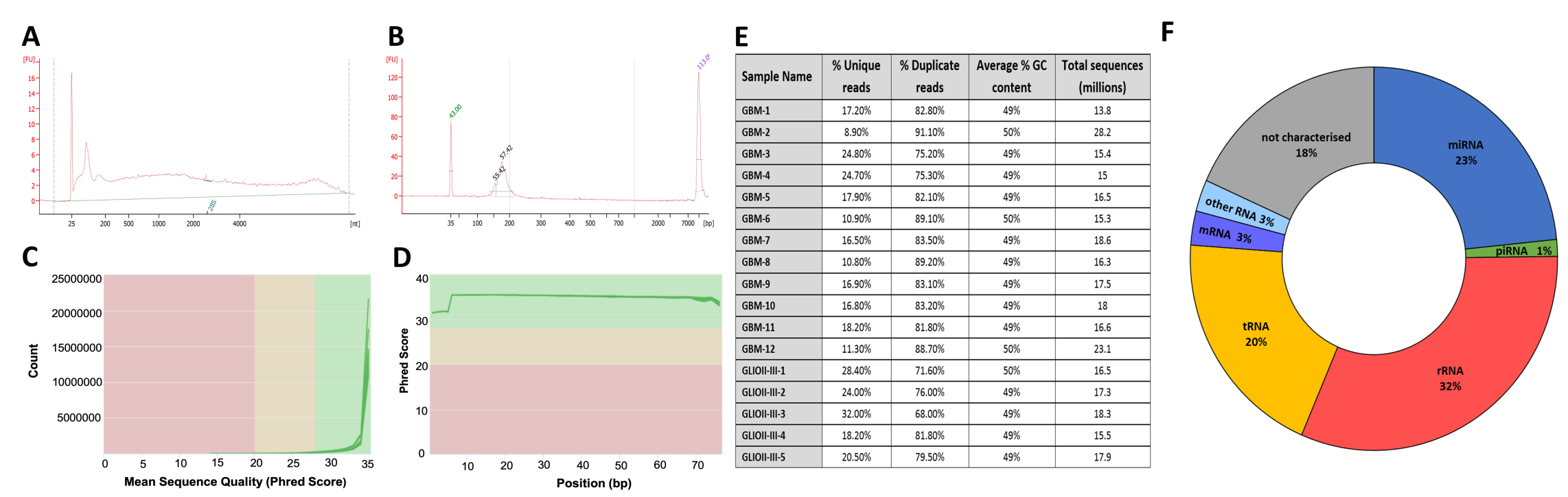


**Figure S1.** Agilent Bioanalyser traces of (A) total RNA extracted from GBM CUSA-EVs and the subsequent (B) cDNA library of the size selected small RNAs. Quality control metrics of small RNA sequenced reads by FastQC showing (C) the number of reads plotted against mean quality scores for all CUSA-EV samples and (D) histogram of mean quality score for each base position in the read for all CUSA EV-samples. Phred scores above 30 (green range) show high quality sequences and base calls. (E) FastQC statistics of the CUSA-EV small non-coding RNA sequencing data. (F) Percentage of the total reads mapped to small non-coding RNAs (miRNA, rRNA, tRNA, piRNA) as well as mRNA and other RNA species.


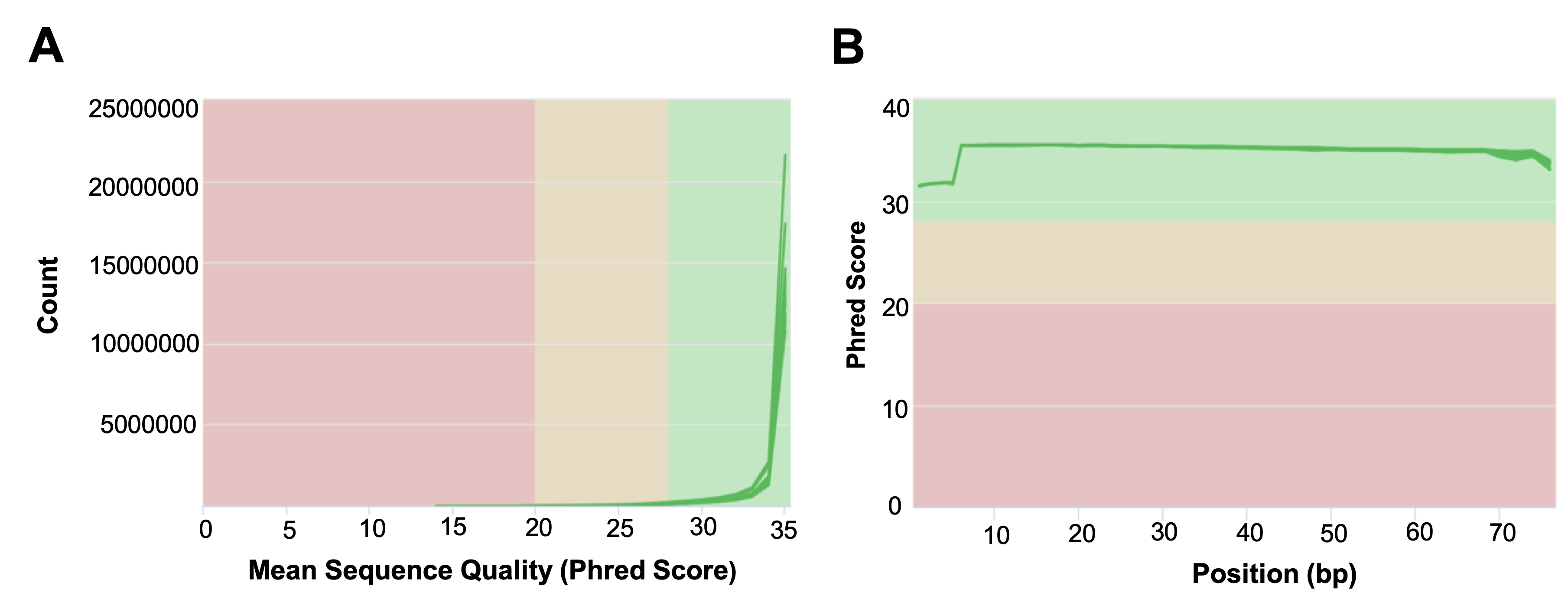

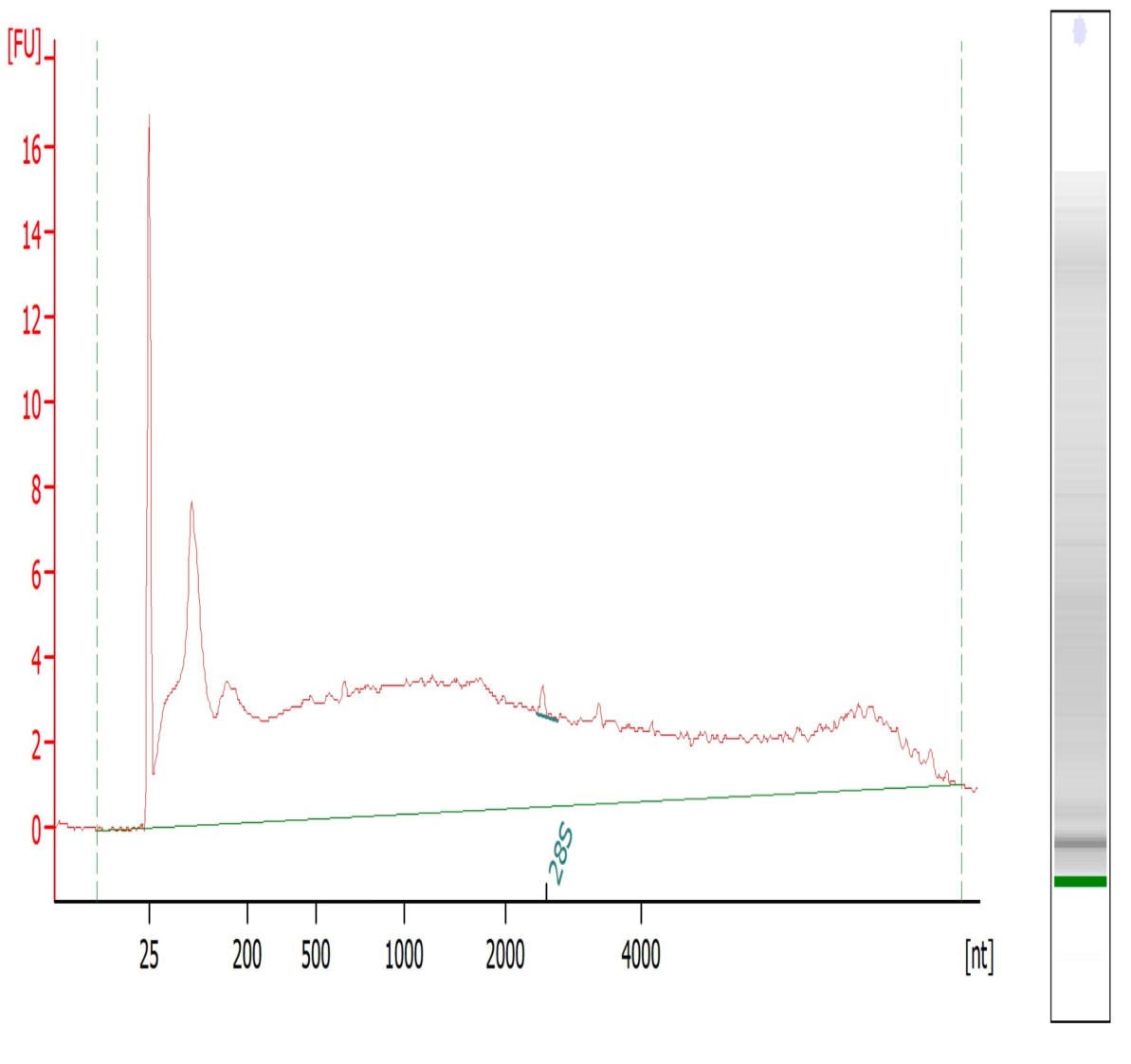

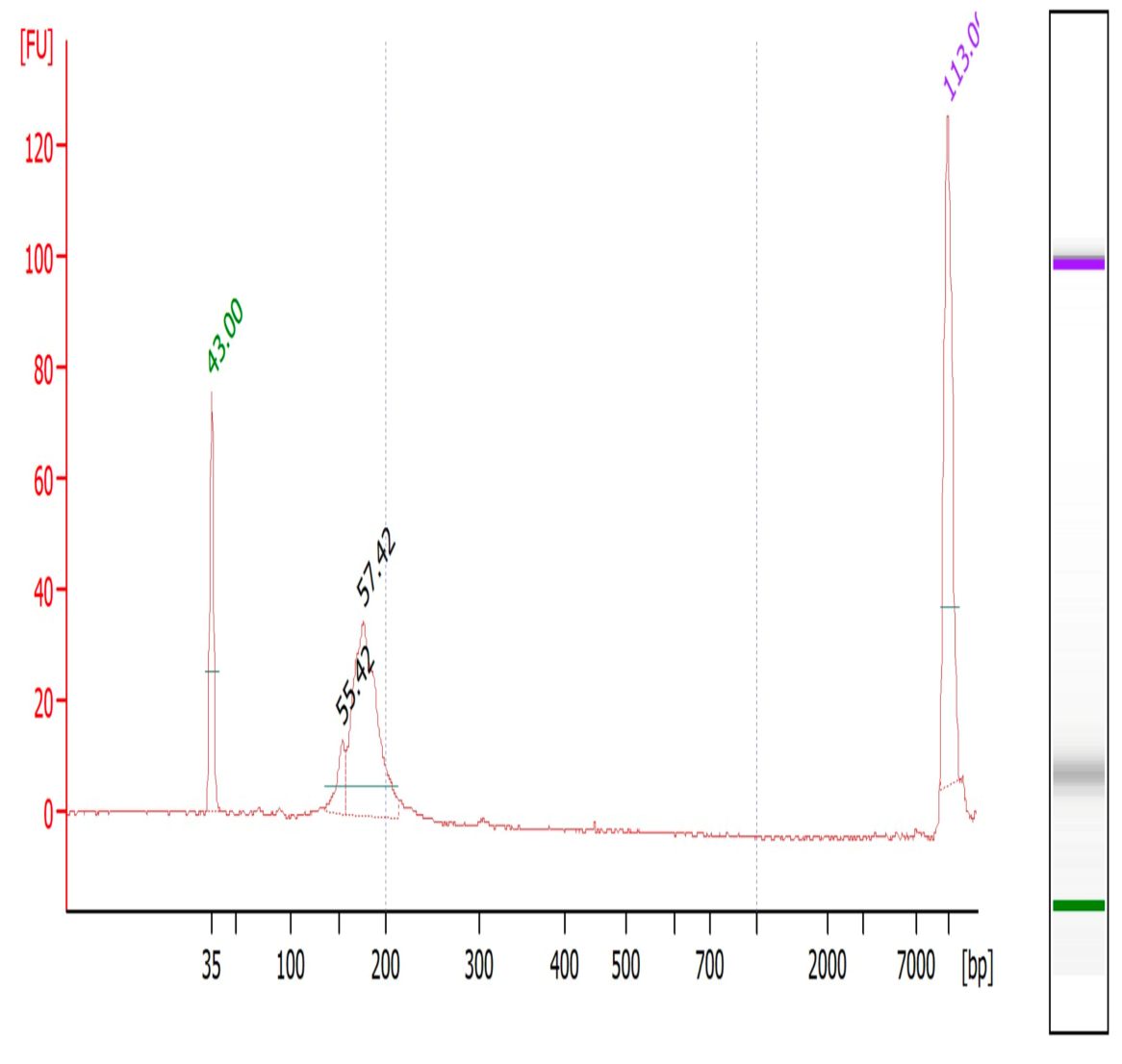

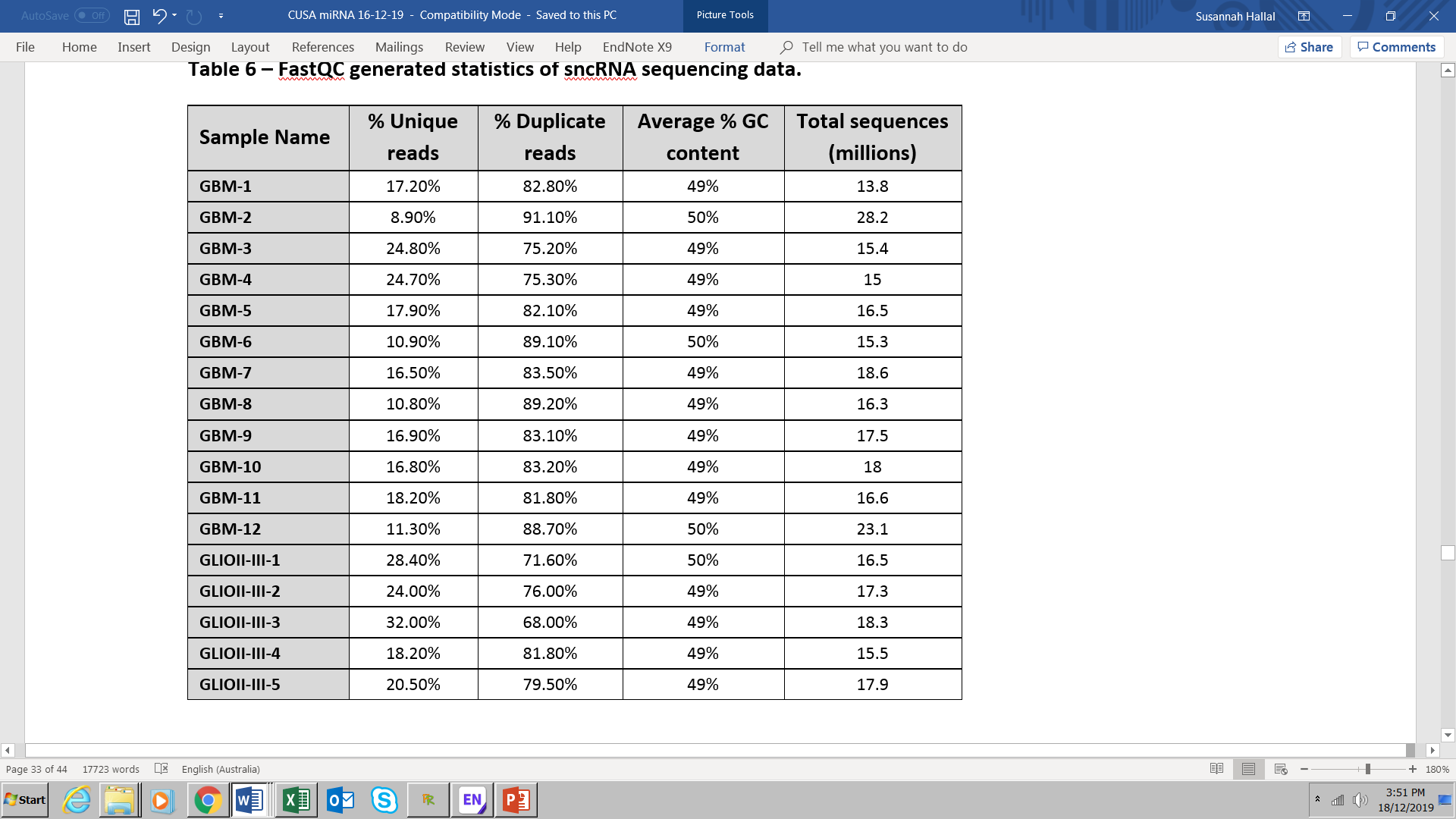


**A**

**B**


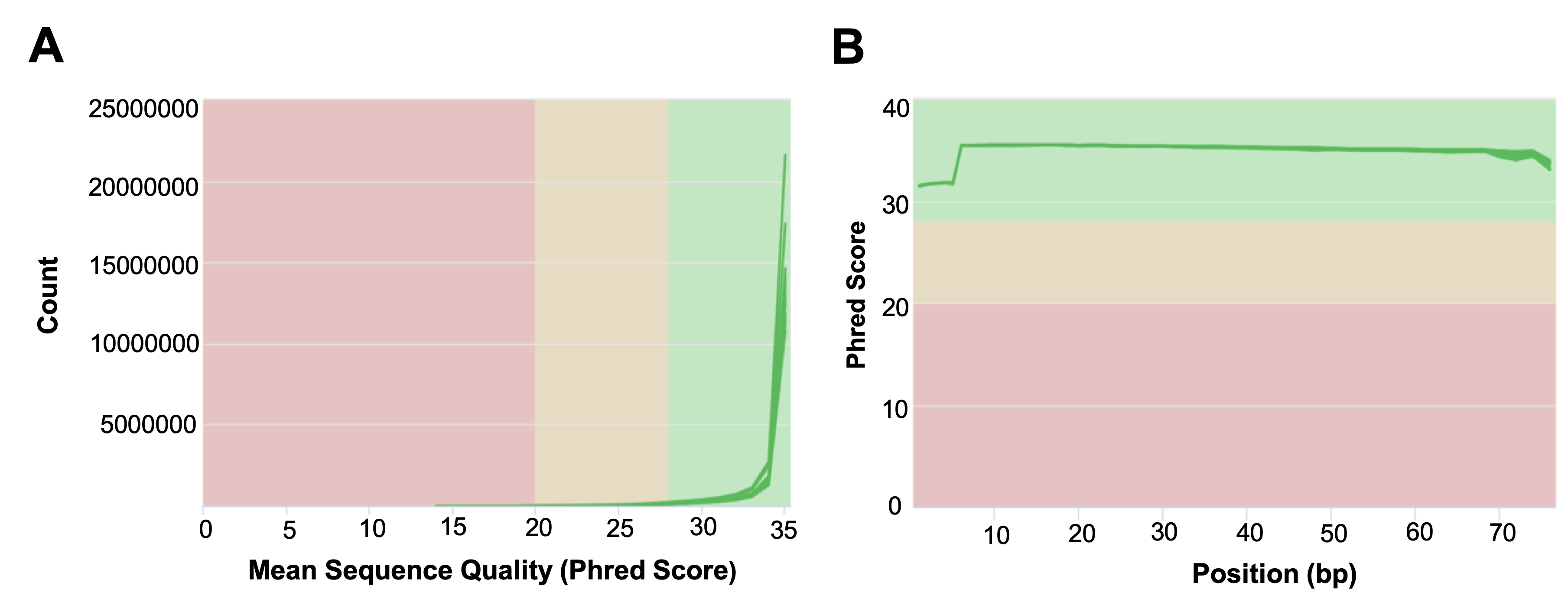


**C**

**D**

**E**


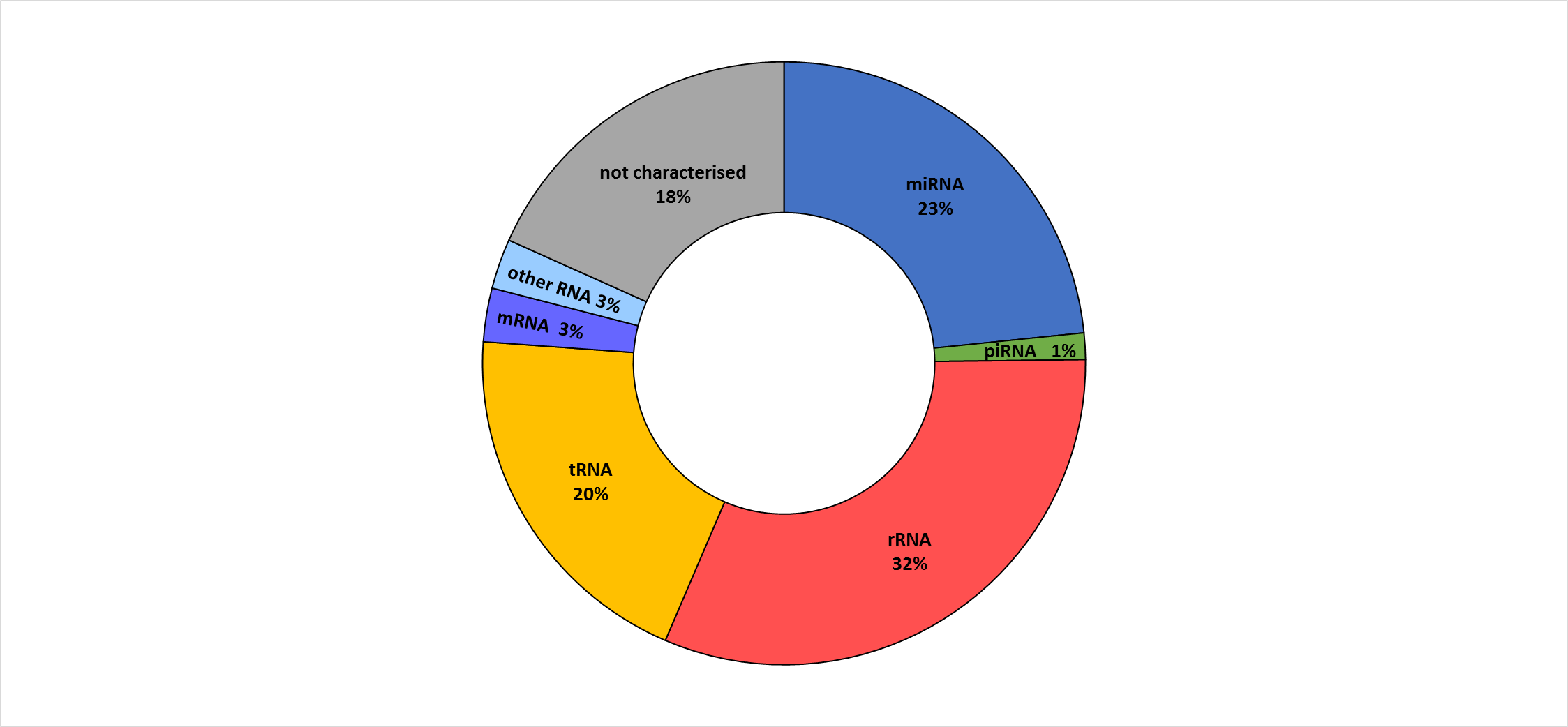


**F**


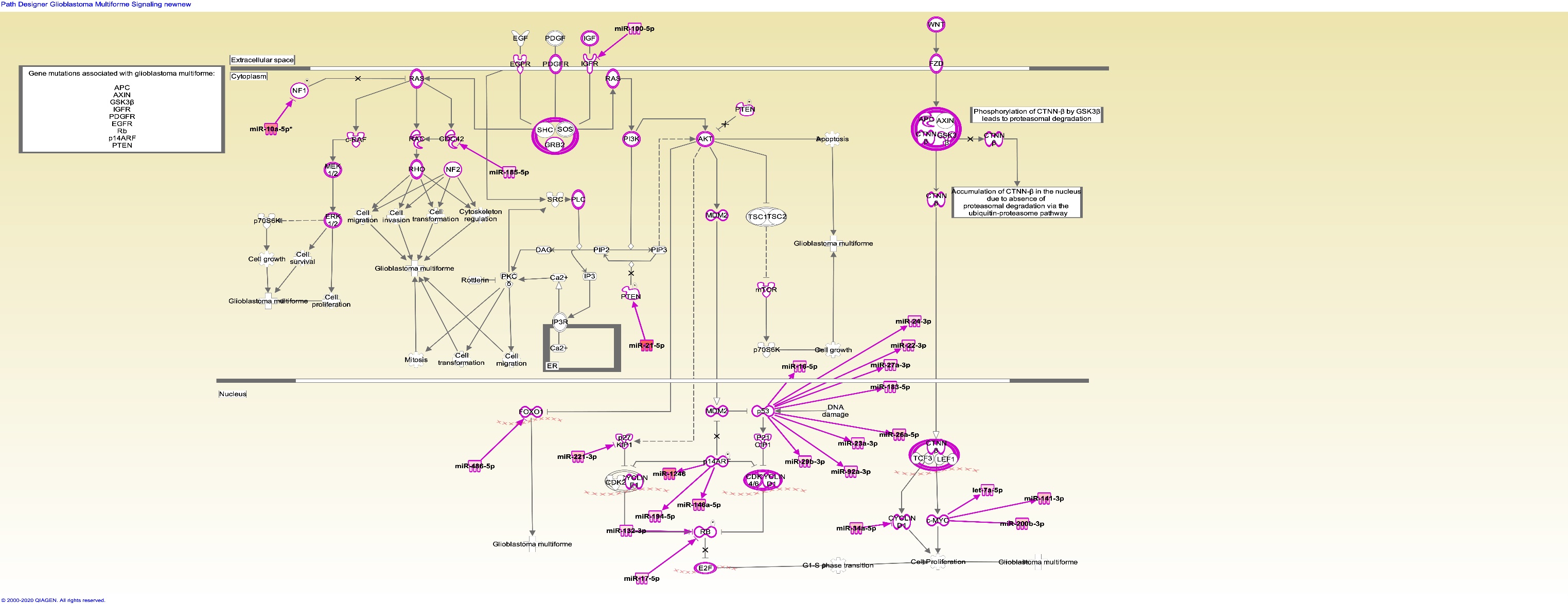

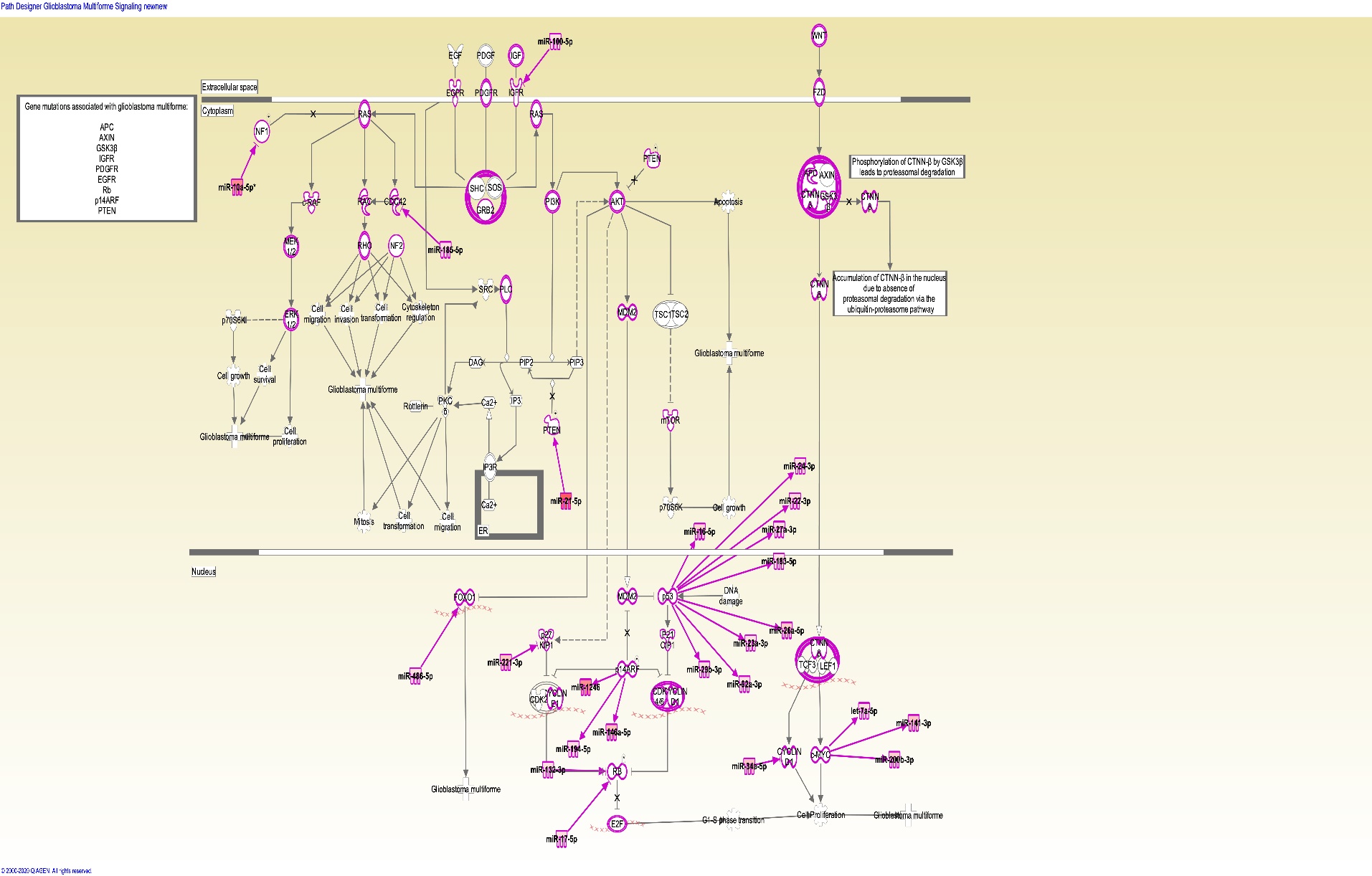


**Figure S2. GBM signalling pathway annotated with molecules targeted and/or affected by differentially expressed miRNA species in GBM CUSA-EVs.** Molecules predicted to be affected are highlighted in magenta. Direct interactions between the affected molecules and significant miRNA species (unadjusted p-value ≤ 0.05, GBM CUSA-EVs relative to GII-III) are overlayed on the pathway; direct upstream miRNA regulation (⇥) and downstream miRNA expression (⟶). The significant miRNAs are shaded in pink, with darker shades representative of higher expression in GBM relative to GII-III CUSA-EVs.
